# Screen-Based Sedentary Behavior, Physical Activity, and Muscle Strength in the English Longitudinal Study of Ageing

**DOI:** 10.1371/journal.pone.0066222

**Published:** 2013-06-03

**Authors:** Mark Hamer, Emmanuel Stamatakis

**Affiliations:** 1 Population Health Domain Physical Activity Research Group, University College London, London, United Kingdom; 2 Department of Epidemiology and Public Health, University College London, London, United Kingdom; 3 School for Physiology, Nutrition and Consumer Sciences, North-West University, Potchefstroom, South Africa; 4 Prevention Research Collaboration, School of Public Health, University of Sydney, Sydney, Australia; Brigham and Women's Hospital, Harvard Medical School, United States of America

## Abstract

**Background:**

Sarcopenia is associated with loss of independence and ill-health in the elderly although the causes remain poorly understood. We examined the association between two screen-based leisure time sedentary activities (daily TV viewing time and internet use) and muscle strength.

**Methods and Results:**

We studied 6228 men and women (aged 64.9±9.1 yrs) from wave 4 (2008-09) of the English Longitudinal Study of Ageing, a prospective study of community dwelling older adults. Muscle strength was assessed by a hand grip test and the time required to complete five chair rises. TV viewing and internet usage were inversely associated with one another. Participants viewing TV ≥6hrs/d had lower grip strength (Men, B = −1.20 kg, 95% CI, −2.26, −0.14; Women, −0.75 kg, 95% CI, −1.48, −0.03) in comparison to <2hrs/d TV, after adjustment for age, physical activity, smoking, alcohol, chronic disease, disability, depressive symptoms, social status, and body mass index. In contrast, internet use was associated with higher grip strength (Men, B = 2.43 kg, 95% CI, 1.74, 3.12; Women, 0.76 kg, 95% CI, 0.32, 1.20). These associations persisted after mutual adjustment for both types of sedentary behaviour.

**Conclusions:**

In older adults, the association between sedentary activities and physical function is context specific (TV viewing vs. computer use). Adverse effects of TV viewing might reflect the prolonged sedentary nature of this behavior.

## Introduction

Age related declines in muscle mass and strength, known as sarcopenia, are a risk factor for health and independence in the elderly. Measures of muscle strength have been associated with morbidity, functional independence and mortality in older populations [1]–[4] even after 25 yrs or more of follow-up. Several studies have documented associations between physical activity and muscle strength tests [5], [6] and direct measures of lean mass [7]–[9] in older individuals, although the findings are not always consistent with some studies observing no associations [10]–[12]. Inconsistencies in the data might be explained by different measures of physical activity (objective versus self-report) but also the failure to consider sedentary activities as an independent domain of behavior.

Prolonged sedentary activities, particularly watching TV, have been associated with a range of adverse health outcomes independently from physical activity [13]–[18]. Thus, sedentary behavior is now considered as a distinct domain of behavior, which may pose a risk to health in its own right. However, most research to date has focused on cardiometabolic and mortality outcomes but the independent role of sedentary behavior in explaining the declines in muscle strength with ageing remains unknown. Previous research suggests that not all types of sedentary behaviors are related with adverse health markers in elderly populations [19], [20], thus it is unclear if the effects are being driven by physiological processes linked to excessive sitting or the specific and broader context of the activity. If associations are only apparent for specific types of sedentary activity this might suggest that residual confounding may be driving the effects. To test the overall hypothesis that excess screen-based sedentary behavior is inversely associated with muscle strength, we examined two types of common sedentary activities in relation to several key functionally relevant tests of physical performance.

## Methods

### Ethics statement

Participants gave full informed written consent to participate in the study and ethical approval was obtained from the London Multi-centre Research Ethics Committee.

### Study sample and procedures

The English Longitudinal Study of Ageing (ELSA) is an ongoing cohort study that contains a nationally representative sample of the English population living in households [21]. The ELSA cohort consists of men and women born on or before 29 February 1952, using multistage stratified probability sampling with postcode sectors selected at the first stage and household addresses selected at the second stage. The data were collected by a team of trained researchers that adhered to strict protocols. For the purposes of the present analyses, data collected at wave 4 (2008-09) were used as this was the first occasion that information on sedentary activities was gathered.

### Sedentary and physical activity

Participants were asked to recall “How many hours of television do you watch on an ordinary day or evening, that is, Monday to Friday?” and “How many hours of television do you normally watch in total over the weekend, that is, Saturday and Sunday?” Average daily time spent watching TV was calculated as {(weekday TV time x 5) + (Weekend TV time)}/7. Daily TV time was categorized into four groups (<2hrs/d; 2 to <4 hrs/d; 4 to <6hrs/d; ≥6 hrs/d). In addition participants were asked if they used a computer for internet or email. We have described the ELSA physical activity measurements in detail previously [22]. In brief, participants were asked how often they took part in three different types of physical activity: vigorous, moderate- and low-intensity physical activity. The response options were: more than once a week, once a week, one to three times a month and hardly ever/never. Physical activity was further categorized into three groups: None (no moderate or vigorous activity on a weekly basis); Moderate activity at least once a week; and Vigorous activity at least once a week.

### Physical strength measures

Physical strength measures included hand grip and a timed chair stand test. Hand grip strength (kg) of the dominant hand was assessed using a hand held dynamometer, with the average of three measures used in the analyses. The intra-class correlation of average measures was high (0.987, 95% CI, 0.987, 0.988). The chair stand test, a measure of lower body strength, assessed the time required to rise from a chair to a full standing position five times with arms folded across the chest, with slower times reflecting worse function. The test incorporated the use of respondent’s own armless, straight backed chair.

### Covariates

Demographic and health-related questions included cigarette smoking (current, previous or non-smoker), frequency of alcohol intake (daily, 5–6/wk, 3–4/wk, 1–2/wk, 1–2/month, once every couple of months, 1–2/year, never) and self-reported chronic illness. Depressive symptoms were assessed using the 8-item Centre of Epidemiological Studies Depression (CES-D) scale, highly validated for use in older adults [23]. Socioeconomic status was based on the last/most recent occupation and categorized into three groups (managerial/ professional; intermediate; routine/manual occupations). We assessed disability based on participants’ responses to questions on perceived difficulties in basic (e.g., difficulty dressing, including putting on shoes and socks) [24] and instrumental (e.g., difficulty preparing a hot meal) activities of daily living [25]. Participants with difficulties in one or more activities were considered to have some degree of disability. Nurses collected anthropometric data (weight, height). Participants’ body weight was measured using Tanita electronic scales without shoes and in light clothing, and height was measured using a Stadiometer with the Frankfort plane in the horizontal position. Body mass index (BMI) was calculated using the standard formulae [weight (kilograms)/height (meters) squared].

### Statistical analyses

In order to examine associations between sedentary behaviors and muscle strength we employed general linear models. The dependent variables were normally distributed. We calculated coefficients and 95% CIs for hand grip strength (kg) and chair stand time (seconds) with reference to the TV viewing category and a binary internet use variable (No/Yes). These analyses were performed separately among men and women due to the well documented differences in muscle strength between sexes [5]. We fitted a series of models, entering covariables using a forward stepwise approach: a basic age-adjusted model, then with additional adjustment for smoking, alcohol, physical activity, social status, disability, chronic illness, depressive symptoms, and BMI. Each model was also mutually adjusted for each type of screen-based sedentary behavior. This modeling strategy was planned *a priori* based on existing data linking these covariates with sedentary behavior and muscle strength [5], [16]. All analyses were conducted using SPSS version 21.

## Results

From the initial 8643 participants that attended wave 4, a total of 8343 participants provided valid data from physical performance tests. Participants that refused or were unable to provide these measures tended to be slightly older than those who consented and had valid measures (70.8±11 vs 68.2±11.3 yrs). A further 2115 participants were excluded due to missing data leaving a final analytic sample of 2845 men and 3383 women (aged 64.9±9.1 yrs). Participants excluded tended to be older (68.2±11.3 yrs, p<0.001), have lower BMI (23.8±11.9 vs. 28.2±5.2 kg/m^2^, p = 0.001), although more depressive symptoms (3.3±1.5 vs. 2.9±1.3, p = 0.001), and a higher prevalence of chronic illness (63.1 vs. 51.4%, p<0.001).

Table 1 presents the characteristics of the sample in relation to TV viewing time. Participants that watched more TV tended to have less healthier profiles in terms of lower physical activity, smoking, greater chronic illness and disability, higher BMI and depressive symptoms. In addition, TV viewing was socially patterned, showing that lower status participants watched more TV. Use of the internet was inversely associated with TV viewing in that participants that watched more TV were less likely to use the internet. Use of the internet was also socially patterned but in the opposite direction to that of TV viewing; a higher proportion of internet users were of higher social occupational class (defined as managerial/ professional) compared with non-users (48.0 vs. 20.6%, p<0.001).

**Table 1:** Characteristics of the sample in relation the TV viewing time.

	<2 hrs/d (n = 655)	2<4 hrs/d (n = 2183)	4<6 hrs/d (n = 1676)	≥ 6 hrs/d (n = 1715)
Age (mean [SD] yrs)	63.8±9.2	64.4 ±9.1	65.6±9.1	65.2±9.0
Men	53.1	48.5	43.1	41.7
Current smokers	6.0	10.2	11.7	18.0
Physically active†	90.0	86.6	80.6	74.9
Regular alcohol intake‡	73.8	69.4	59.2	57.2
Lowest social status*	17.7	26.2	39.1	53.8
Chronic illness	44.8	47.5	53.8	56.3
Disability	16.6	16.6	21.9	30.4
Obese (BMI ≥30 kg/m^2^)	17.6	26.2	34.3	38.5
Users of internet	76.6	69.7	54.5	43.3

Data presented as percentages unless otherwise stated.

† defined as moderate or vigorous activity at least once per week.

‡ defined as alcohol intake at least once per week.

* Defined as routine/manual occupations.

As expected, men recorded higher grip strength (38.7±9.5 vs. 22.9±6.4 kg, p<0.001) and a faster time to complete 5 chair rises (10.9±3.7 vs. 11.4±4.1 sec, p<0.001) compared with women. Other variables that were consistently and independently associated with the physical performance tests included physical activity, disability, chronic illness, depressive symptoms and social occupational class (see Tables S1 and S2). Higher BMI was associated with higher grip strength but a slower time to complete 5 chair rises, which likely reflects the contribution of higher lean and fat mass that is advantageous only for non-weight bearing tests. TV viewing time was inversely associated with grip strength in both men and women (see Figures S1 and S2), and although these associations were somewhat attenuated they still persisted after adjustment for covariables (Tables 2 and 3). Participants viewing TV ≥6hrs/d had lower grip strength (Men, coefficient  = −1.20 kg, 95% CI, −2.26, −0.14; Women, −0.75 kg, 95% CI, −1.48, −0.03) in comparison to <2hrs/d TV, after multivariate adjustments. The same pattern of results was obtained when TV time was modelled as a continuous variable (Men, fully adjusted B = −0.16, 95% CI, −0.23, −0.08; Women, B =  −0.06, 95% CI, −0.11, −0.01). In contrast, internet use was associated with greater grip strength (Men, coefficient  = 2.43 kg, 95% CI, 1.74, 3.12; Women, 0.76 kg, 95% CI, 0.32, 1.20). These associations persisted after mutual adjustment for each type of sedentary activity. Higher TV viewing was associated with a slower time to complete 5 chair rises in age adjusted models although the effect estimates were attenuated to the null after multivariate adjustments. Internet usage was associated with faster chair rise time although significant effects only persisted among men in multivariate models (see Table 2).

**Table 2:** The association between TV time, internet use and muscle strength indicators in men (N = 2845).

Sedentary exposure	N	Age adjustedB* (95% CI)	MultivariateB* (95% CI)	Age adjustedB* (95% CI)	MultivariateB* (95% CI)
*TV time*		*Hand grip (Kg)*		*Chair stand time (secs)*	
<2 hrs/d	349	Reference	Reference	Reference	Reference
2<4 hrs/d	1055	−0.54 (−1.54, 0.46)	−0.58 (−1.55, 0.39)	−0.04 (−0.48, 0.39)	−0.20 (−0.62, 0.22)
4<6 hrs/d	724	−0.73 (−1.79, 0.32)	−0.27 (−1.30, 0.77)	0.24 (−0.22, 0.70)	−0.25 (−0.70, 0.20)
≥6 hrs/d	717	−2.47 (−3.52, −1.41)	−1.20 (−2.26, −0.14)	0.65 (0.18, 1.12)	−0.08 (−0.54, 0.39)
*p-trend*		<0.001	0.076	0.001	0.62
*Internet usage*					
No	1007	Reference	Reference	Reference	Reference
Yes	1838	3.25 (2.58, 3.92)	2.43 (1.74, 3.12)	−0.84 (−1.14, −0.54)	−0.44 (−0.75, −0.13)
*p-trend*		<0.001	<0.001	<0.001	0.005

**Multivariate model** includes adjustments for age, smoking, physical activity, alcohol, social class, disability, chronic illness, body mass index, CES-D score, and mutually for TV time or internet use.

* General linear model coefficients; coefficients indicate mean differences (in muscle strength markers) between each screen time group and the reference category.

**Table 3:** The association between TV time, internet use and muscle strength in women (N = 3383).

Sedentary exposure	N	Age adjustedB* (95% CI)	MultivariateB* (95% CI)	Age adjustedB* (95% CI)	MultivariateB* (95% CI)
*TV time*		*Hand grip (kg)*		*Chair stand time (secs)*	
<2 hrs/d	306	Reference	Reference	Reference	Reference
2<4 hrs/d	1127	−0.47 (−1.19, 0.25)	−0.41 (−1.10, 0.29)	−0.04 (−0.46, 0.54)	−0.14 (−0.64, 0.34)
4<6 hrs/d	952	−1.07 (−1.81, −0.34)	−0.65 (−1.37, 0.07)	0.53 (0.02, 1.04)	−0.04 (−0.54, 0.45)
≥6 hrs/d	998	−1.54 (−2.26, −0.81)	−0.75 (−1.48, −0.03)	0.71 (0.20, 1.22)	−0.04 (−0.54, 0.47)
*p-trend*		<0.001	0.173	<0.001	0.90
*Internet usage*					
No	1541	Reference	Reference	Reference	Reference
Yes	1842	1.55 (1.12, 1.98)	0.76 (0.32, 1.20)	−0.79 (−1.10, −0.49)	−0.17 (−0.48, 0.13)
*p-trend*		<0.001	0.001	<0.001	0.268

**Multivariate model** includes adjustments for age, smoking, physical activity, alcohol, social class, disability, chronic illness, body mass index, CES-D score, and mutually for TV time or internet use.

* General linear model coefficients; coefficients indicate mean differences (in muscle strength markers) between each screen time group and the reference category.

In sensitivity analyses, missing values were imputed (SPSS multiple imputation procedure) based on maximum likelihood estimates although a similar pattern of results was obtained. For example, the association between TV time (modelled as a continuous variable) and hand grip strength using imputed data was similar to the original results (Men, fully adjusted B = −0.12, 95% CI, −0.14, −0.09; Women, B =  −0.04, 95% CI, −0.06, −0.03).

## Discussion

The aim of this study was to examine associations between two key leisure time screen-based sedentary activities (daily TV viewing time and internet use) and common tests of physical performance in a sample of community dwelling older adults. Several previous studies have observed associations between physical activity and muscle strength tests [5], [6] or lean mass [7]–[9] although few have examined the independent contribution of sedentary behavior. We confirmed the results of previous studies by demonstrating associations between physical activity and muscle strength that were apparent at relatively low levels of moderate activity (Tables S1 and S2). We also observed associations for screen-based sedentary behavior and physical performance although they appeared to be context-specific in that TV viewing was associated with lower muscle strength but the opposite effects were observed for internet use. Our data are consistent with a previous study in young adults that demonstrated inverse associations between TV time and muscular fitness tests including trunk extension and flexion strength [26]. The effect sizes we observed may have clinical relevance given that a 1 kg increase in grip strength was associated with a pooled reduction in mortality risk of 3% in a recent meta-analysis [3].

These data may be interpreted in several ways. Firstly, given that these two sedentary behaviors were strongly socially patterned but in opposite directions suggest that the results might merely reflect residual confounding effects of social status that cannot be fully captured by the occupational social class measure that we adjusted our analyses for. We have repeatedly shown that combining different socioeconomic indicators can considerably improve the prediction of leisure-time screen-based sedentary behaviour [27]. However, it might be argued that our measure of internet use was crude and we were unable to examine dose-response associations. It is possible that people watch TV for more prolonged periods of time as data from the UK time use survey showed that computer users spend, on average, 2 hrs/d on a computer [28]. Several lines of evidence suggest that TV viewing carries its own health risks over and above sitting. Firstly we have shown discrepancies in results when using objectively assessed total sedentary time compared with self-reported TV time to predict cardiometabolic outcomes [19]. Other studies have shown discrepancies between workplace sitting and TV viewing when predicting cardiovascular risk factors [29].

The associations we observed for screen-based sedentary behaviour were largely confined to hand grip strength. This is perhaps not surprising because chair rising is a far more complex test that not only involves strength but also neuromuscular control. Hand grip is a simple isometric test of upper body muscle strength. Previous data from middle aged British adults suggested that physical activity had stronger protective effects on handgrip strength in men than in women [5]. The patterns of association between sedentary behavior, physical activity, and muscle strength in ELSA were broadly similar in men and women, thus the reasons for these discrepancies remain unclear.

Our study has some limitations. We did not assess all types of sedentary behaviors and therefore our results cannot be generalized to total sedentary time. Nevertheless, TV viewing is arguably the most prevalent form of sedentary activity in the elderly [28] and our data confirm previous reports as over a quarter of our sample reported watching TV over 6 hrs/d. There were some differences in the characteristics of participants included and excluded from our analyses. In light of these biases the estimates presented herein might reflect a conservative estimate of the true associations, although results from sensitivity analyses using imputed data largely replicated our original analyses. Our data are based on self-report that might have introduced bias, thus we cannot discount the possibility of residual confounding. Nevertheless, we have previously demonstrated the validity of measures such as self-reported morbidity in ELSA [30]. Since these analyses were cross-sectional we cannot discount the possibility of reverse causation in that poorer general physical conditioning (as reflected by lower muscle strength) causes people to spend more time sedentary and not the reverse. Indeed, a recent study in middle aged adults demonstrated that BMI at baseline was prospectively associated with greater TV viewing at follow-up but not the converse [20]. Despite these limitations, our study also has some notable strengths. These include the use of a large national sample of community-dwelling men and women and the ability to adjust for a wide range of potentially important confounding factors, including behavioral, social and clinical variables.

In conclusion, we observed associations between two key screen-based sedentary behaviors and muscle strength independently of physical activity, although the relationships appeared to be context specific. Our results suggest that TV viewing carries its own health risks in older age over and above the sitting it entails.

## Supporting Information

Figure S1 Unadjusted mean grip strength in relation to TV viewing.
(DOCX)

Figure S2 Scatter plot of TV viewing against grip strength (upper panel) and chair rises time (lower panel).
(DOCX)

Table S1 The association between covariables and muscle strength in men (N = 2845).
(DOCX)

Table S2 The association between covariables and muscle strength in women (N = 3383).
(DOCX)
