# Supplementary figures and images for: Screen-Based Sedentary Behavior, Physical Activity, and Muscle Strength in the English Longitudinal Study of Ageing
Source: PLoS One. 2013 Jun 3;8(6):e66222. doi: 10.1371/journal.pone.0066222 (PMC3670922; doi:10.1371/journal.pone.0066222)

**Figure S1.** Unadjusted mean grip strength in relation to TV viewing.


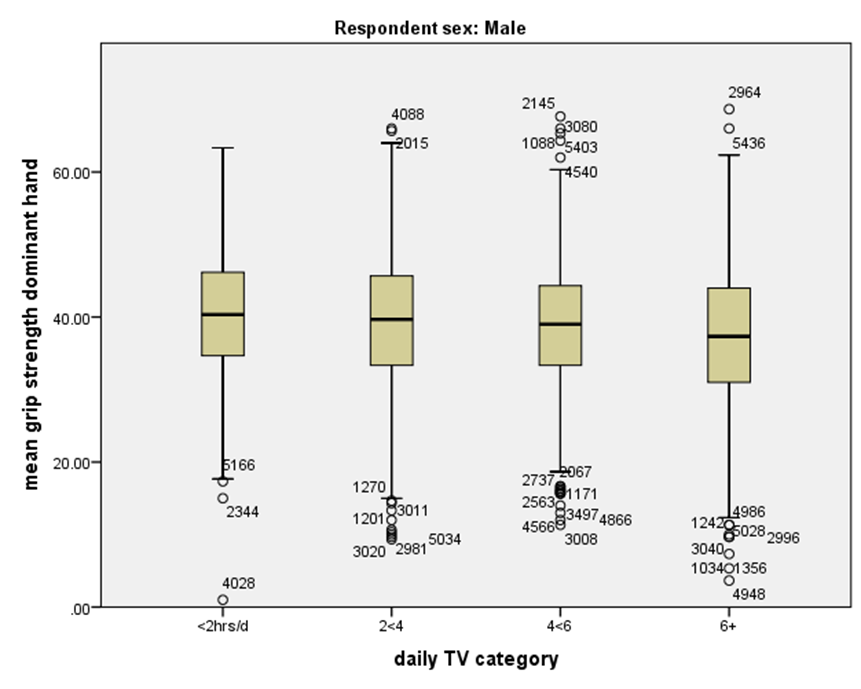


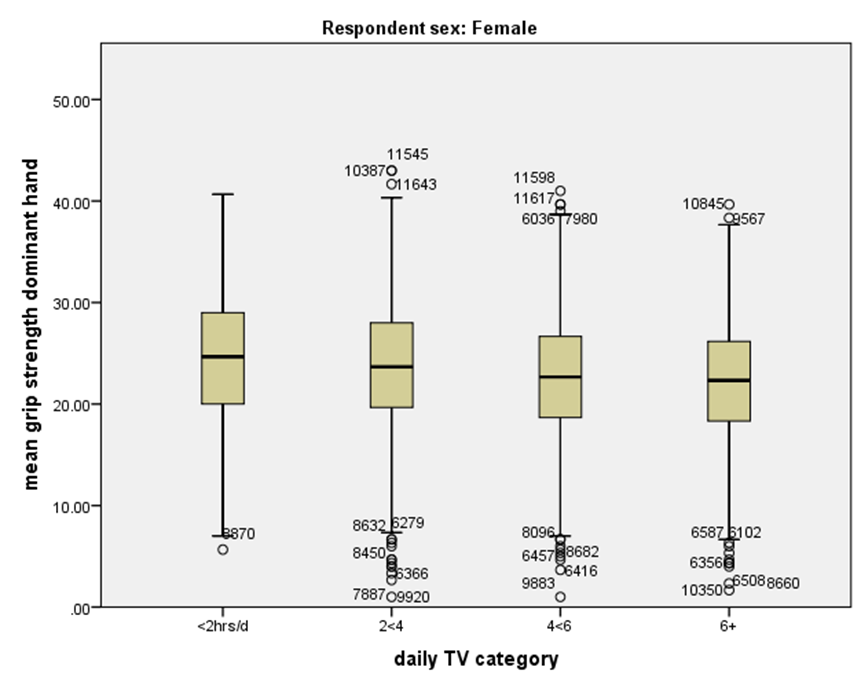

Supplement: Figure S1 — Unadjusted mean grip strength in relation to TV viewing. (DOCX) [file pone.0066222.s001.docx]
