# Supplementary material for: Screen-Based Sedentary Behavior, Physical Activity, and Muscle Strength in the English Longitudinal Study of Ageing
Source: PLoS One. 2013 Jun 3;8(6):e66222. doi: 10.1371/journal.pone.0066222 (PMC3670922; doi:10.1371/journal.pone.0066222)

**Figure S2.** Scatter plot of TV viewing against grip strength (upper panel) and chair rises time (lower panel)


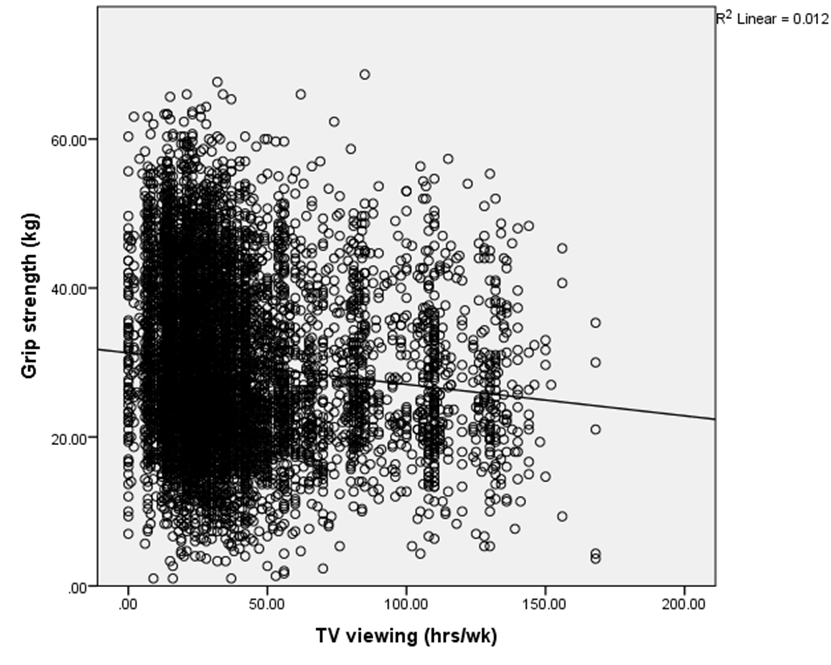


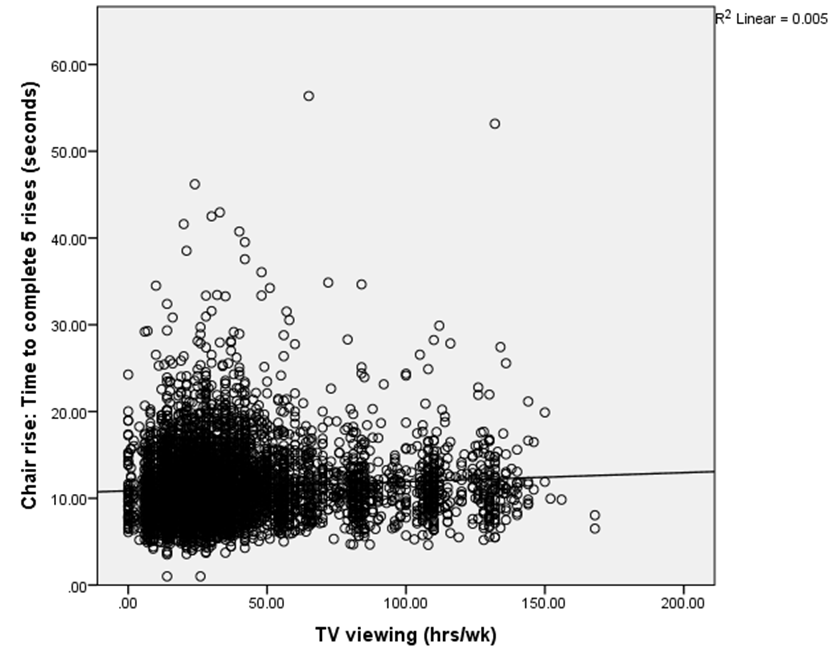

Supplement: Figure S2 — Scatter plot of TV viewing against grip strength (upper panel) and chair rises time (lower panel). (DOCX) [file pone.0066222.s002.docx]
