# Supplementary material for: Screen-Based Sedentary Behavior, Physical Activity, and Muscle Strength in the English Longitudinal Study of Ageing
Source: PLoS One. 2013 Jun 3;8(6):e66222. doi: 10.1371/journal.pone.0066222 (PMC3670922; doi:10.1371/journal.pone.0066222)
Supplement: Table S1 — The association between covariables and muscle strength in men (N = 2845). (DOCX) [file pone.0066222.s003.docx]

**Table S1** The association between covariables and muscle strength in men (N=2845).

| **Variable** | **N** | **Multivariate model**  **B (95% CI)** | **Multivariate model**  **B (95% CI)** |
| --- | --- | --- | --- |
| *Physical activity* |  | *Hand grip (Kg)* | *Chair stand time (secs)* |
| Inactive | 409 | Reference | Reference |
| Moderate | 1395 | 1.56 (0.64, 2.49) | -1.18 (-1.61, -0.75) |
| Vigorous | 1041 | 2.89 (1.89, 3.88) | -1.84 (-2.30, -1.39) |
| *p-trend* |  | <0.001 | <0.001 |
| *Smoking* |  |  |  |
| Yes | 336 | Reference | Reference |
| No | 2510 | 0.29 (-0.67, 1.25) | -0.58 (-1.01, -0.14) |
| *p-trend* |  | 0.55 | 0.01 |
| *Alcohol* |  |  |  |
| Rarely/never | 315 | Reference | Reference |
| Once/month | 410 | 0.74 (-0.45, 1.93) | -0.75 (-1.29, -0.21) |
| 1<5/wk | 1255 | 0.91 (-0.10, 1.93) | -0.54 (-1.01,- 0.08) |
| ≥ 5/wk | 866 | 1.37 (0.31, 2.43) | -0.63 (-1.11, -0.14) |
| *p-trend* |  | 0.08 | 0.039 |
| *Disability* |  |  |  |
| Yes | 570 | Reference | Reference |
| No | 2276 | 1.94 (1.12, 2.77) | -1.41 (-1.79, -1.03) |
| *p-trend* |  | <0.001 | <0.001 |
| *Chronic illness* |  |  |  |
| Yes | 1454 | Reference | Reference |
| No | 1392 | 0.80 (0.17, 1.44) | -0.49 (-0.76, -0.21) |
| *p-trend* |  | 0.013 | 0.001 |
| *Depression (CES-D≥4)* |  |  |  |
| Yes | 500 | Reference | Reference |
| No | 2346 | 1.17 (0.35, 2.00) | -0.40 (-0.77, -0.03) |
| *p-trend* |  | 0.005 | 0.034 |
| *Body mass index* |  |  |  |
| 15 - 25 | 625 | Reference | Reference |
| <25<30 | 1408 | 2.19 (1.42, 2.95) | 0.08 (-0.26, 0.41) |
| ≥30 | 813 | 3.22 (2.26, 4.09) | 0.41 (0.03, 0.79) |
| *p-trend* |  | <0.001 | 0.062 |
| *Social occupational class* |  |  |  |
| Managerial/ professional | 1260 | Reference | Reference |
| Intermediate | 591 | 0.16 (-0.64, 0.95) | 0.04 (-0.30, 0.39) |
| Manual/routine | 982 | -0.84 (-1.55, -0.13) | 0.42 (0.11, 0.73) |
| *p-trend* |  | 0.06 | 0.05 |

**Multivariate model** includes mutual adjustment for all variables presented plus sedentary behaviors
