# Supplementary material for: Screen-Based Sedentary Behavior, Physical Activity, and Muscle Strength in the English Longitudinal Study of Ageing
Source: PLoS One. 2013 Jun 3;8(6):e66222. doi: 10.1371/journal.pone.0066222 (PMC3670922; doi:10.1371/journal.pone.0066222)
Supplement: Table S2 — The association between covariables and muscle strength in women (N = 3383). (DOCX) [file pone.0066222.s004.docx]

**Table S2** The association between covariables and muscle strength in women (N=3383).

| **Variable** | **N** | **Multivariate model**  **B (95% CI)** | **Multivariate model**  **B (95% CI)** | |
| --- | --- | --- | --- | --- |
| *Physical activity* |  | *Hand grip (Kg)* | *Chair stand time (secs)* | |
| Inactive | 702 | Reference | Reference | |
| Moderate | 1682 | 0.69 (0.18, 1.20) | -0.59 (-0.96, -0.22) | |
| Vigorous | 999 | 1.81 (1.22, 2.40) | -1.42 (-1.83, -1.00) | |
| *p-trend* |  | <0.001 | <0.001 | |
| *Smoking* |  |  |  | |
| Yes | 428 | Reference | Reference | |
| No | 2955 | -0.37 (-0.95, 0.20) | -0.52 (-0.94, -0.10) | |
| *p-trend* |  | 0.205 | 0.014 | |
| *Alcohol* |  |  |  | |
| Rarely/never | 777 | Reference | Reference | |
| Once/month | 756 | 0.001 (-0.56, 0.56) | 0.04 (-0.46, 0.34) | |
| 1<5/wk | 1232 | 0.37 (-0.15, 0.89) | -0.14 (-0.50, 0.23) | |
| ≥ 5/wk | 618 | 1.03 (0.42, 1.64) | -0.41 (-0.84, 0.02) | |
| *p-trend* |  | 0.002 | 0.141 | |
| *Disability* |  |  |  | |
| Yes | 784 | Reference | Reference | |
| No | 2599 | 1.60 (1.10, 2.11) | -1.45 (-1.81, -1.08) | |
| *p-trend* |  | <0.001 | <0.001 | |
| *Chronic illness* |  |  |  | |
| Yes | 1745 | Reference | Reference | |
| No | 1638 | 0.99 (0.59, 1.39) | -0.83 (-1.11, -0.56) | |
| *p-trend* |  | <0.001 | <0.001 | |
| *Depression (CES-D≥4)* |  |  |  | |
| Yes | 935 | Reference | Reference | |
| No | 2448 | 1.16 (0.72, 1.60) | -0.76 (-1.07, -0.45) | |
| *p-trend* |  | <0.001 | <0.001 | |
| *Body mass index* |  |  |  | |
| 15 - 25 | 1058 | Reference | Reference | |
| <25<30 | 1211 | 0.31 (-0.15, 0.37) | 0.11 (-0.20, 0.43) | |
| ≥30 | 1114 | 0.98 (0.49, 1.47) | 0.76 (0.42, 1.10) | |
| *p-trend* |  | <0.001 | <0.001 | |
| *Social occupational class* |  |  | |  |
| Managerial/ professional | 1030 | Reference | | Reference |
| Intermediate | 1030 | -0.34 (-0.82, 0.14) | | 0.38 (0.05, 0.71) |
| Manual/routine | 1284 | -0.53 (-1.01, -0.05) | | 0.47 (0.14, 0.80) |
| *p-trend* |  | 0.19 | | 0.015 |

**Multivariate model** includes mutual adjustment for all variables presented plus sedentary behaviors
